# Indoor metals pollution and metabolic outcomes among Italian children

**DOI:** 10.1093/eurpub/ckac129.525

**Published:** 2022-10-25

**Authors:** S Renzetti, M Schito, L Borgese, P Cirelli, G Cagna, A Patrono, M Peli, R Ranzi, RG Lucchini, D Placidi

**Affiliations:** 1 Università degli Studi di Brescia, Brescia, Italy; 2 Florida International University, Miami, USA

## Abstract

**Background:**

Through this study, we aimed to test the association between the exposure to metals measured in indoor dust and metabolic outcomes among children living in areas with environmental exposure to metals.

**Methods:**

The project “Health impacts of environmental exposure to airborne pollutants in the sites of Brescia and Taranto, Italy: increase knowledge to address preventive intervention of local and global relevance” (ISEIA) enrolled 130 children aged 6 to 13 years (51.5% females) resident from pregnancy in highly industrialized areas of the Brescia province, Northern Italy. Metabolic outcomes including BMI, fasting blood glucose and blood creatinine were measured. The concentration of a mixture of 10 metals in indoor dust was determined through an X-ray fluorescence portable analyzer (p-XRF). Linear regressions and Weighted Quantile Sum (WQS) regression were applied to test for the association between metal exposure and metabolic outcomes. All models were adjusted for age, gender, socio-economic status and area of residence.

**Results:**

A significant association was observed between Cd and blood creatinine (β = 0.01; 95%CI=0.001, 0.02; p-value=0.028) when considering each individual metal separately in the model. WQS regression showed a positive significant association between the mixture of metals and fasting glucose (β = 0.87; 95%CI 0.14, 1.61; p-value=0.023) identifying Mn, Cr and Cu as the elements with the higher weights, while a marginally significant association was found between the metal mixture and blood creatinine (β = 0.01; 95%CI=-0.001, 0.02; p-value=0.075) where Cd and Ti showed the highest weight.

**Conclusions:**

We assessed the potential association between exposure to metals in indoor household dust and blood glucose and creatinine. Our results contribute to clarifying the role of metal exposure in the burden of non-communicable diseases although further studies are needed to better understand the relationship between metal exposure and metabolism.

**Key messages:**

• The metal mixture in indoor household dust is associated with an increase in fasting glucose.

• Cd concentration in indoor household dust is associated with an increase in blood creatinine.

